# Supplementary material for: Tailoring the component of protein corona via simple chemistry
Source: Nat Commun. 2019 Oct 4;10:4520. doi: 10.1038/s41467-019-12470-5 (PMC6778128; doi:10.1038/s41467-019-12470-5)
Supplement: Supplementary file 1 — Suplementary Information [file 41467_2019_12470_MOESM1_ESM.pdf]

Supplementary Information

Tailoring the component of protein corona via simple chemistry

Lu *et al.*

Contents:

Supplementary Figures 1 – 7

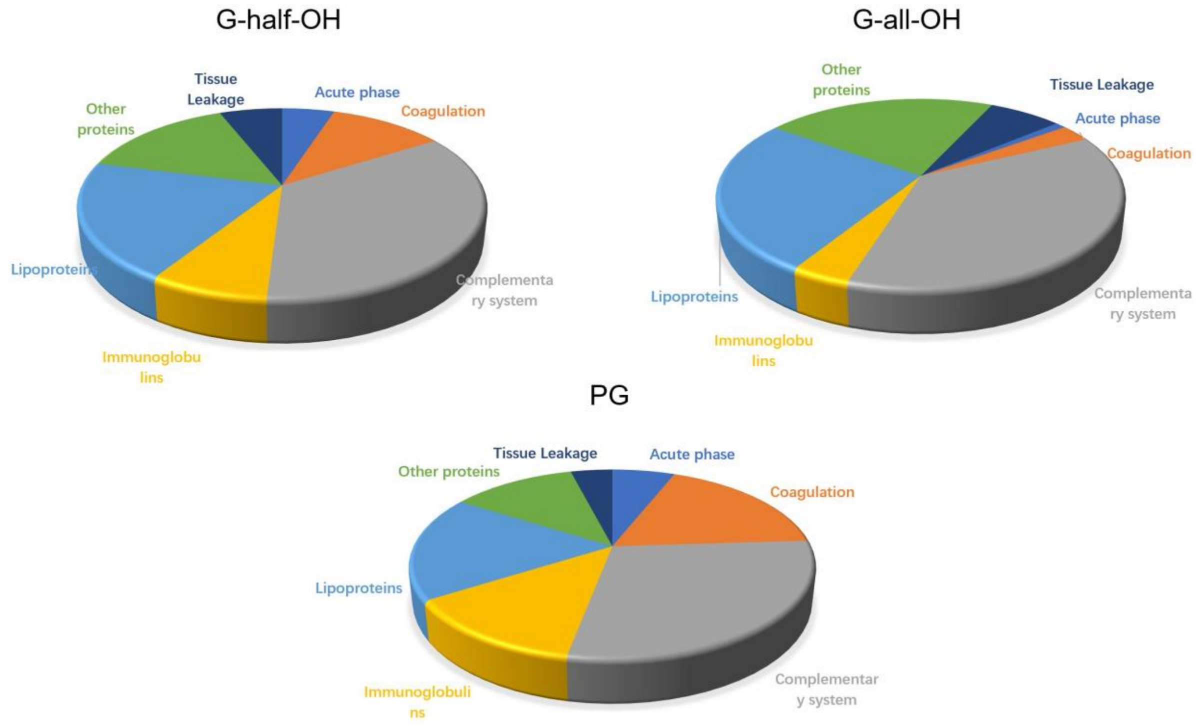

Supplementary Figure 1. Proteomic analysis showing the composition of protein corona encasing G-half/all-OH and PG incubated with serum. Of note, the pie-chart was generated based on the number of recognized proteins belonging to each of the seven categories including lipoproteins, coagulation, tissue leakage, immunoglobulins, acute phase, complementary system, and other types.

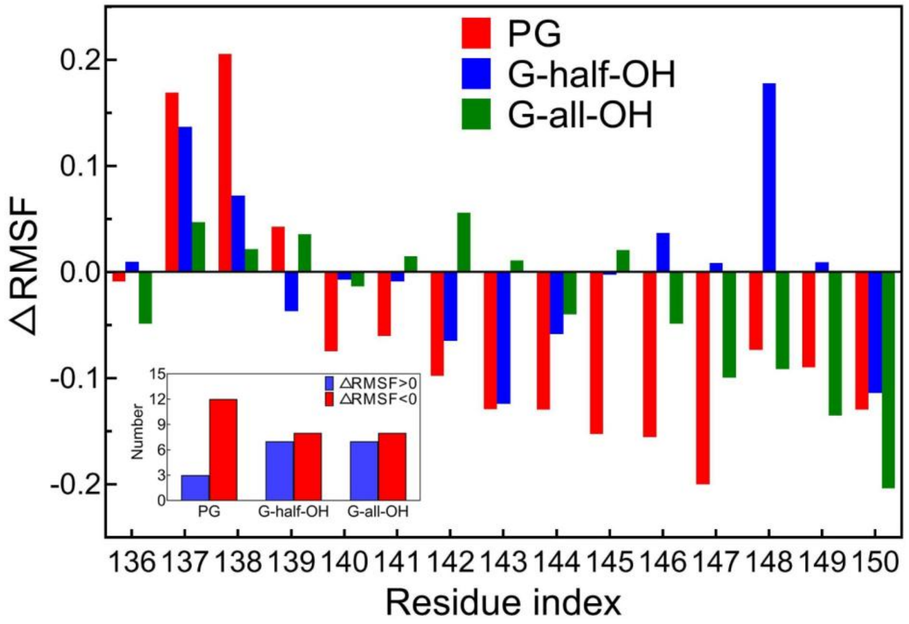

Supplementary Figure 2. The RMSF (root mean square fluctuation) difference parameter ΔRMSF of the active/binding site (residues 136-150) of ApoE when adsorbed onto graphene sheet with different surface modifications (PG, G-half-OH, G-all-OH). ΔRMSF is used to characterize the change in flexibility of residues (compared to that in free state), i.e., $\Delta RMSF=(RMSF_{gra}-RMSF_{free})/RMSF_{free}$, where $RMSF_{free}$ is the RMSF of the C_α atom of residues on the ApoE in free state (i.e., in solution), and $RMSF_{gra}$ is the RMSF of the C_α atom of residues on the ApoE adsorbed on PG/G-half-OH/G-all-OH. A positive value of ΔRMSF (i.e., ΔRMSF >0) indicates an increase in the flexibility of residues, while a negative value (i.e., ΔRMSF <0) indicates a decrease in the flexibility. The inserted table summarizes the increased or decreased numbers of ΔRMSF in all cases. The more the increased number (of ΔRMSF), the more active the binding site is; the more the decreased number (of ΔRMSF), the less active the binding site is.

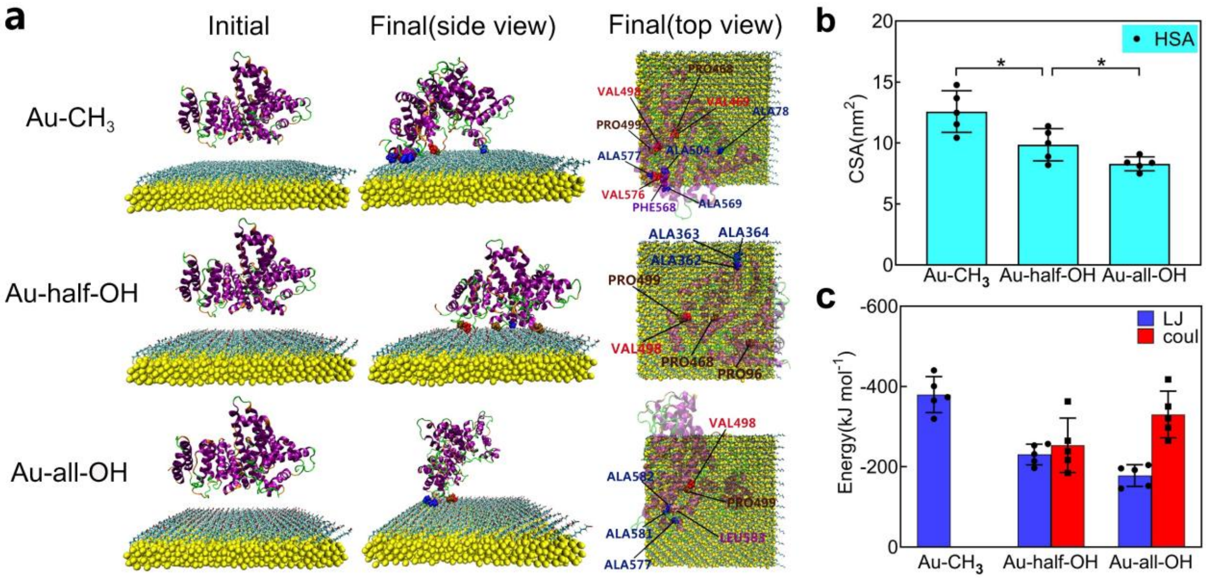

Supplementary Figure 3. The simulation results of the interaction of HSA with the Au nanoparticle. **a** Typical snapshots of the initial and final structures of the adsorption of HSA onto the surface (yellow) with different surface modifications. From the side and top views, hydrophobic residues within 0.5 nm distance from the surface are represented as CPK spheres. Water molecules and ions are omitted for clarity. **b**, **c** The contact surface area (CSA) and the Lennard-Jones (LJ) and Coulomb (Coul) interaction energy between HSA and the Au nanoparticle with different surface modifications, respectively. Data are presented as mean ± s.e.m. (n=5). P values were calculated using multiple t tests (*$p<0.05$).

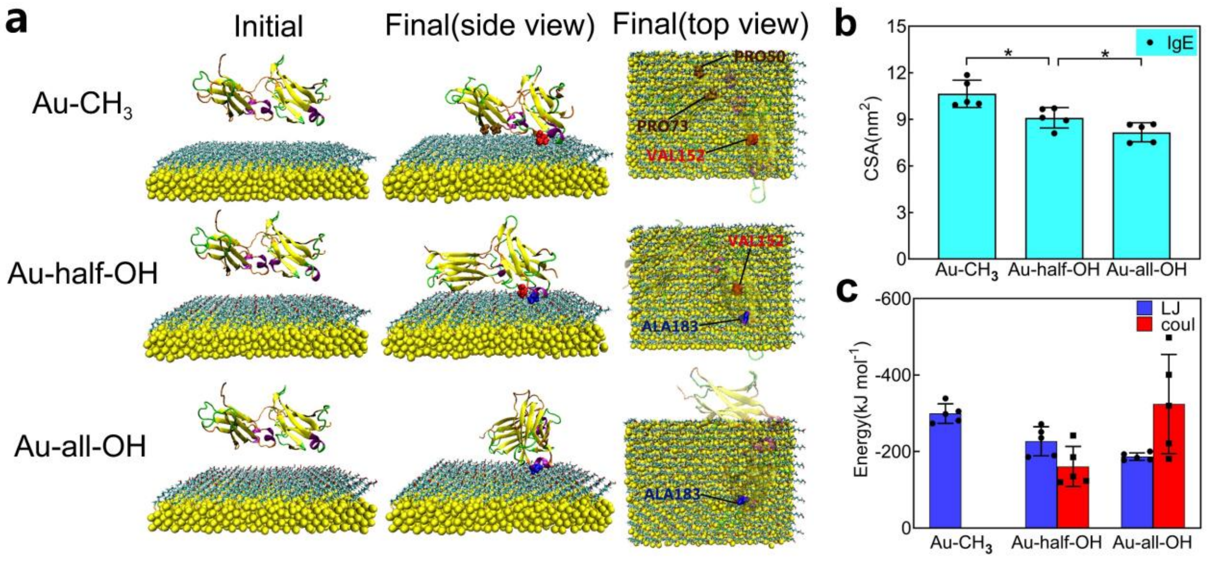

Supplementary Figure 4. The simulation results of the interaction of IgE with the Au nanoparticle. **a** Typical snapshots of the initial and final structures of the adsorption of IgE onto the surface (yellow) with different surface modifications. From the side and top views, hydrophobic residues within 0.5 nm distance from the surface are represented as CPK spheres. Water molecules and ions are omitted for clarity. **b**, **c** The contact surface area (CSA) and the Lennard-Jones (LJ) and Coulomb (Coul) interaction energy between IgE and the Au nanoparticle with different surface modifications, respectively. Data are presented as mean ± s.e.m. (n=5). P values were calculated using multiple t tests (*$p<0.05$).

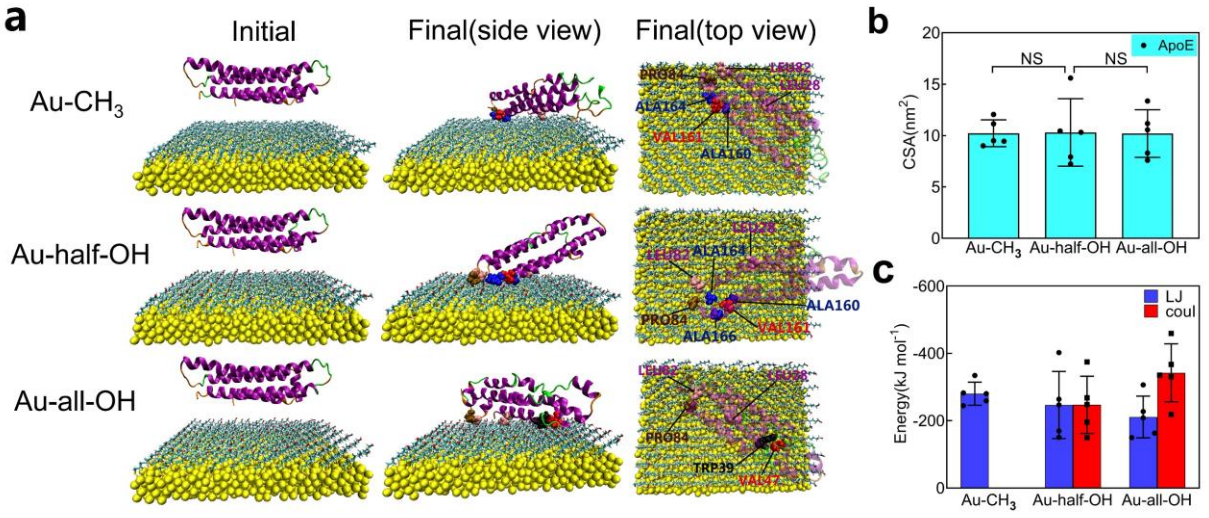

Supplementary Figure 5. The simulation results of the interaction of ApoE with the Au nanoparticle. **a** Typical snapshots of the initial and final structures of the adsorption of ApoE onto the surface (yellow) with different surface modifications. From the side and top views, hydrophobic residues within 0.5 nm distance from the surface are represented as CPK spheres. Water molecules and ions are omitted for clarity. **b**, **c** The contact surface area (CSA) and the Lennard-Jones (LJ) and Coulomb (Coul) interaction energy between ApoE and the Au nanoparticle with different surface modifications, respectively. Data are presented as mean ± s.e.m. (n=5). P values were calculated using multiple t tests (*$p<0.05$).

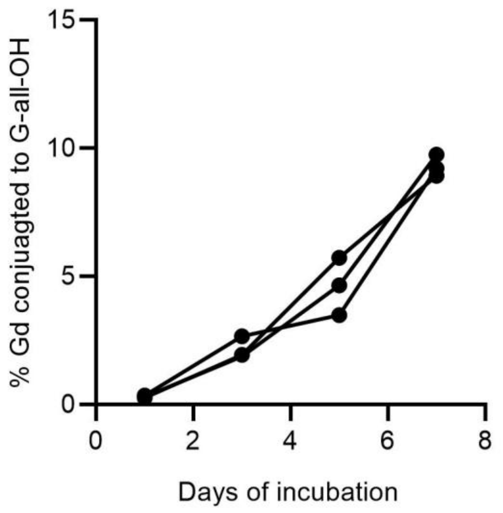

Supplementary Figure 6. Integrity of Gd-tag. The Gd-labeled graphene was incubated with fresh mouse serum, and the content of free Gd formed as a consequence of premature detachment was analyzed by inductively coupled plasma-mass spectrometer (ICP-MS). Three independent tests were conducted during a period of 7 days, with each result shown individually.

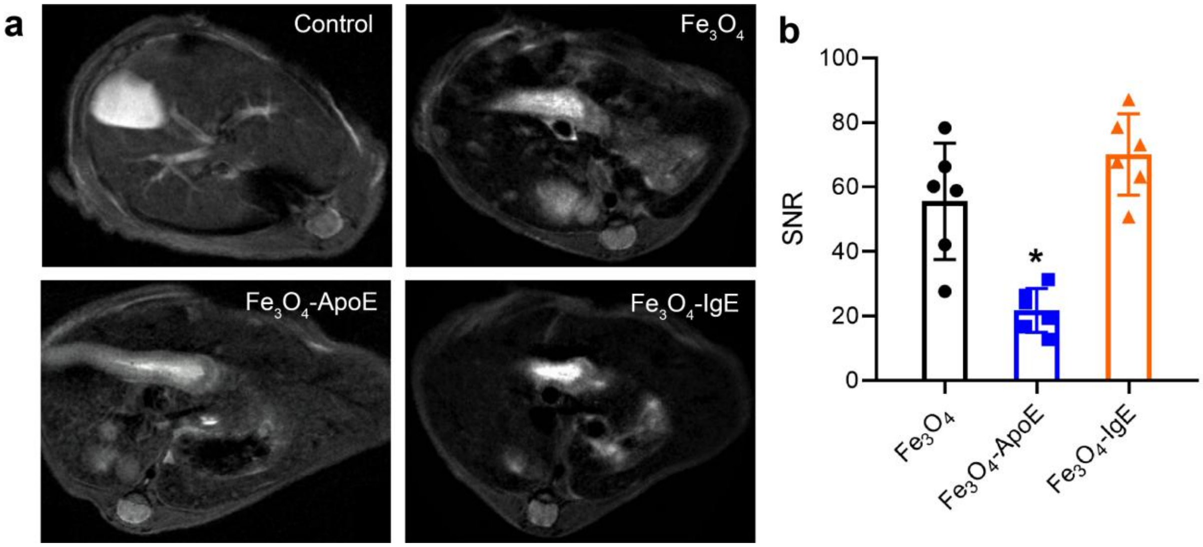

Supplementary Figure 7. a T_2-weighted magnetic resonance imaging results. Images of liver tissue of mice received different nanomaterials were collected at 8 h post the injection. b Quantification of imaging results by using the signal-to-noise ratio (SNR) to reflect the content of enhancement in each case. Data are presented as mean ± s.e.m. (n=5). P values were calculated using multiple t tests (*$p<0.05$).
